# Diversity in Alzheimer’s disease drug trials: The importance of eligibility criteria

**DOI:** 10.1002/alz.12433

**Published:** 2021-09-30

**Authors:** Sanne Franzen, Jade Emily Smith, Esther van den Berg, Monica Rivera Mindt, Rozemarijn L. van Bruchem-Visser, Erin L. Abner, Lon S. Schneider, Niels D. Prins, Ganesh M. Babulal, Janne M. Papma

**Affiliations:** 1 Department of Neurology, Erasmus MC University Medical Center, Rotterdam, the Netherlands; 2 Department of Psychology and Latin American Latino Studies Institute, Fordham University, The Bronx, New York, USA; 3 Department of Neurology, The Icahn School of Medicine at Mount Sinai, New York, New York, USA; 4 Department of Internal Medicine, Erasmus MC University Medical Center, Rotterdam, the Netherlands; 5 Sanders-Brown Center on Aging and Alzheimer’s Disease Center, University of Kentucky, Lexington, Kentucky, USA; 6 College of Public Health, Department of Epidemiology, University of Kentucky, Lexington, Kentucky, USA; 7 College of Public Health, Department of Biostatistics, University of Kentucky, Lexington, Kentucky, USA; 8 Keck School of Medicine of the University of Southern California, Los Angeles, California, USA; 9 Alzheimer Center, Department of Neurology, VU University Medical Center, Amsterdam, the Netherlands; 10 Brain Research Center, Amsterdam, the Netherlands; 11 Department of Neurology and Knight Alzheimer’s Disease Research Center, Washington University School of Medicine, St. Louis, Missouri, USA; 12 Department of Psychology, University of Johannesburg, Johannesburg, South Africa

**Keywords:** clinical trial, clinical trial protocols, cultural diversity, ethnic groups, phase II, phase III, randomized controlled trials

## Abstract

**Introduction:**

To generalize safety and efficacy findings, it is essential that diverse populations are well represented in Alzheimer’s disease (AD) drug trials. In this review, we aimed to investigate participant diversity in disease-modifying AD trials over time, and the frequencies of participant eligibility criteria.

**Methods:**

A systematic review was performed using Medline, Embase, the Cochrane Library, and Clinicaltrials.gov, identifying 2247 records.

**Results:**

In the 101 included AD trials, participants were predominantly White (median percentage: 94.7%, interquartile range: 81.0–96.7%); and this percentage showed no significant increase or decrease over time (2001–2019). Eligibility criteria such as exclusion of persons with psychiatric illness (78.2%), cardiovascular disease (71.3%) and cerebrovascular disease (68.3%), obligated caregiver attendance (80.2%), and specific Mini-Mental State Examination scores (90.1%; no significant increase/decrease over time) may have led to a disproportionate exclusion of ethnoracially diverse individuals.

**Discussion:**

Ethnoracially diverse participants continue to be underrepresented in AD clinical trials. Several recommendations are provided to broaden eligibility criteria.

## 1 | INTRODUCTION

Although ethnoracially diverse individuals are at an increased risk of developing Alzheimer’s disease (AD) dementia,^1–4^ these populations are systematically underrepresented in AD clinical trials.^5–7^ To generalize safety and efficacy findings from drug trials to the general population, it is essential to include a diverse population, as differences in pharmacokinetics and pharmacodynamics across diverse populations may impact treatment effect and safety;^8,9^ for instance, drug metabolism rates may differ.^6^ The lack of diversity among clinical trial participants is often attributed to enrolling and retaining practices, such as recruitment strategies that do not account for factors that play a role in diverse populations, including mistrust and worry because of historical racism in medical research or the possibility of injury or complications.^10^

Although recruitment factors should be taken into consideration, other explanations need to be considered as well, especially because a number of studies have indicated that people from underrepresented populations may be equally willing to participate in health research.^11,12^ One important potential cause is that there are inherent features of AD-clinical trial eligibility criteria that lead to a disproportionate and systemic exclusion of underrepresented populations.^13,14^ In 1997, Schneider et al.^14^ demonstrated that applying the eligibility criteria of typical AD clinical trials to a Californian memory clinic population led to a systematic underrepresentation of people who are older, female, ethnoracially diverse, lower educated, and less wealthy; they provided several suggestions to improve provisional eligibility, such as a wider range of allowed scores on the Mini-Mental State Examination (MMSE^15^) or by allowing more patients with (mild) behavioral and psychological symptoms to participate.

This systematic review aims to take a closer look at diversity in clinical trials and eligibility criteria. The first goal was to investigate the level of participant diversity in AD clinical trials in the decades after the publication of Schneider et al.^14^ The second goal was to identify which eligibility criteria have been used and how these eligibility criteria were defined. Third, we aimed to assess whether the use of criteria related to cognitive and neuropsychiatric instruments such as the MMSE have changed over time, as these were highlighted by Schneider et al.^14^ as particularly problematic. Last, we will discuss how some eligibility criteria may have affected diversity levels in AD clinical trials.

## 2 | METHODS

### 2.1 | Search strategy

We performed a systematic review using Medline (which includes PubMed), Embase, the Cochrane Library, and ClinicalTrials.gov, without restrictions on the year of publication or location of the trial. Search terms included different terms for AD and mild cognitive impairment (MCI), terms referring to disease-modifying drugs, terms related to amyloid beta (A*β*) and tau, and different terms for phase II and phase III trials (for the complete lists of the search terms used, see Text S1 in supporting information). Studies were included up to December 2019. Two independent authors screened all collected study data (JS and SF). Disagreement was resolved by a consensus agreement together with JMP. The PRISMA (Preferred Reporting Items for Systematic Reviews and Meta-Analyses) guidelines^16^ were followed, except for an assessment of the risk of bias—this step was omitted, as the aim was not to review or summarize the treatment effect reported in the included clinical trials.

### 2.2 | Eligibility criteria

To be included in the review:

1. The study needed to be a planned, ongoing, completed, or early terminated phase II or phase III drug trial for patients with AD dementia, prodromal AD (early AD stage 3^17^), or amnestic MCI (aMCI).
2. The experimental drug was a disease-modifying treatment. Disease-modifying was defined as targeting the pathogenic steps in the A*β* or tau pathways. This includes passive vaccination, monoclonal antibodies, agents disrupting accumulation or aggregation, and agents increasing clearance. As no agreed-upon standards are currently available that definitively delineate which drugs are considered disease-modifying, drug mechanisms were confirmed by consulting relevant literature (e.g., Galimberti and Scarpini^18^) and examining trial features (e.g., outcomes measuring amyloid clearance).

To adequately capture recent developments; collate study results; and provide a clearly delineated, concise set of recommendations, we focused on a homogeneous set of trials and excluded several other types of trials and study populations from this review. First, we excluded studies focusing on other forms of dementia. Second, we excluded AD prevention trials (e.g., lifestyle intervention trials) and studies in preclinical AD (early AD stages 1–2^17^) as these types of trials present with unique challenges and eligibility criteria. Third, we excluded studies focused on symptomatic treatment of AD, including studies of acetylcholinesterase inhibitors—tacrine, donepezil, rivastigmine, and galantamine—and memantine. Fourth, we excluded trials investigating herbal and dietary treatments (e.g., vitamin supplements, olive oil, huperzine). Conference abstracts, dissertations, comments, editorials, book chapters, white papers, and reviews were also excluded.

### 2.3 | Data extraction

For each included study, all available study protocol sources—that is, published papers or National Clinical Trial (NCT) database, European Union Drug Regulating Authorities Clinical Trial Database (EudraCT), and Australian New Zealand Clinical Trial Registry (ANZCTR) clinical trial registrations—identified in the search were used for data extraction. When available, the year that the study was first posted, the study phase, the investigational drug, the inclusion and exclusion criteria, the number of recruited participants, and participant demographics were recorded. Information was compiled from all available sources to create the most complete account of each study’s design and study sample.

### 2.4 | Data analysis

Participant eligibility criteria were divided into three main categories: (1) criteria related to medical conditions; (2) criteria related to undergoing specific study procedures, such as neuropsychological tests and brain scans; and (3) criteria based on diagnostic tests and questionnaire outcomes. Analyses were mostly descriptive. We used Cochran-Armitage trend tests (using the CATT package in R) to assess trends over time for binary variables, that is, whether a criterion was used in the trial or not. Spearman correlations were used to analyze associations between the study start year and continuous variables.

## 3 | RESULTS

**FIGURE 1:**
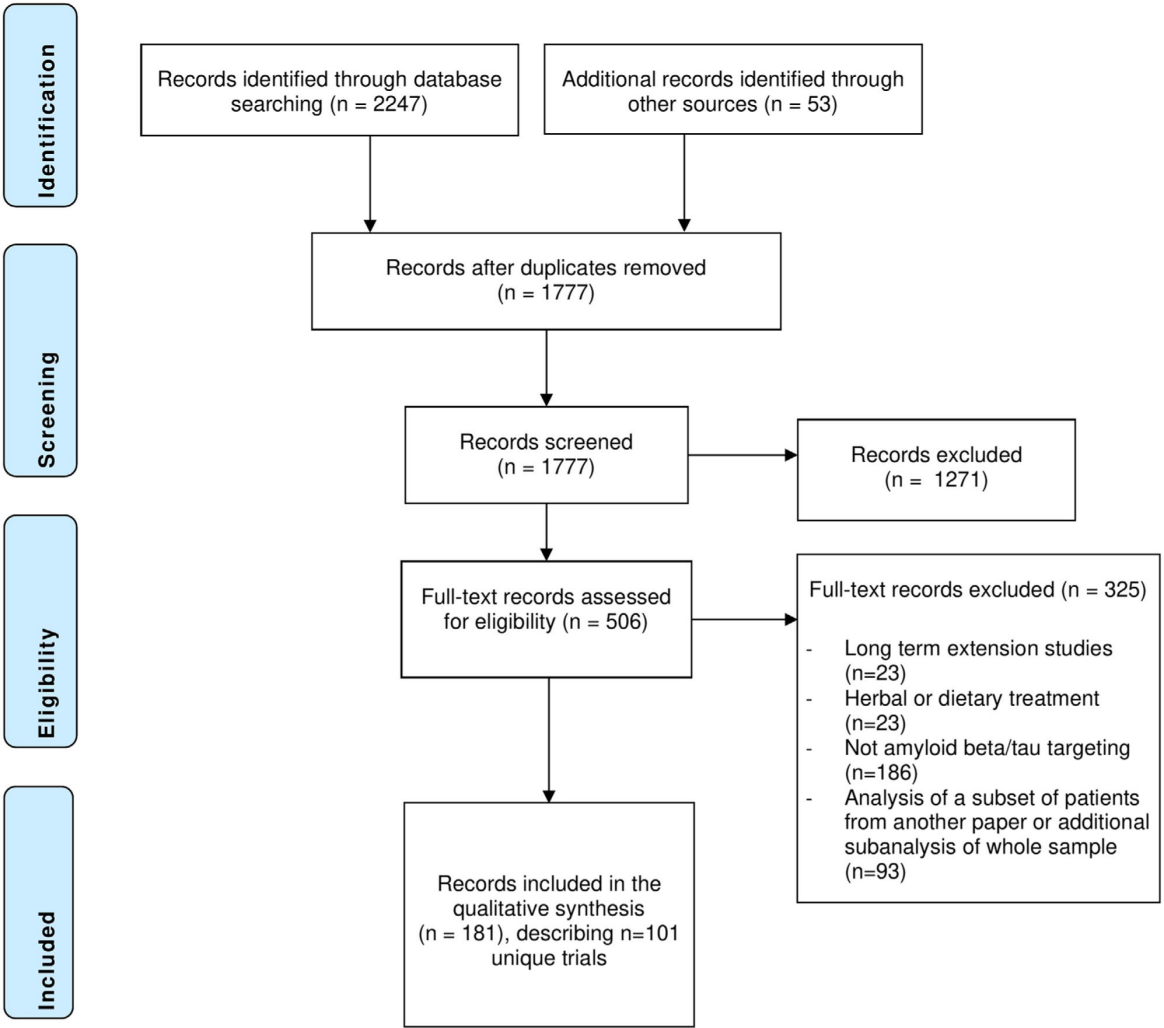
Results of database searches and selection process

We identified 2247 records. The review process is summarized in the PRISMA flowchart in Figure 1. After deduplication, 1777 records remained; these records were screened on title and abstract. If the topic of the abstract fell within the criteria, but there was insufficient information on drug mechanism and/or trial phase, we reviewed the full text. A total of 506 records (clinical trial registrations or papers) were assessed in full for eligibility. A total of 17 NCT registrations, 35 EudraCT registrations, and one ANZCTR registration linked to published papers were retrieved manually. For three studies for which a published paper was available, we could not identify a clinical trial registration.

A total of 101 trials were included in this review. We extracted information about these trials from 181 unique papers and clinical trial registrations, as well as from 21 full protocols that were attached to the included papers or clinical trial registrations. The full protocols were not publicly available for the remaining trials. The sample consisted of 67 phase II trials and 34 phase III trials, investigating 47 different drugs. The studies covered 2001 to 2019, during which 79 studies had finished recruitment, and 22 studies had not yet commenced or were registered as active/recruiting. A listing of the included papers and clinical trial registration numbers is provided in Table S1 in supporting information. Several of the eligibility criteria were more prevalent in studies for which a full protocol was available as opposed to studies for which a full protocol was not available (see Text S2 in supporting information).

### 3.1 | Diversity in clinical trial participants

Of the 101 trials, most had one or more study site(s) in North America (79.2%) or Europe (60.4%), and less frequently Asia (36.6%), or Oceania (32.7%); even fewer trials included study sites in South America (14.9%) or Africa (6.9%). Race/ethnicity data of the enrolled participants was available for less than half of the clinical trials (46 studies, 45.5%). Of these trials, 10 (9.9%) reported only the percentage of White participants without specifying percentages for any other ethnoracial groups, and four (4.0%) included White participants only. Race/ethnicity data was available for 58.2% (46/79) of the studies that were registered as completed or early terminated. When looking specifically at trials for which a published paper was available, 75.5% reported any race/ethnicity data (40/53). Different race/ethnicity categorizations were used across studies. Trials in Clinicaltrials.gov often reported race and/or ethnicity according to the National Institutes of Health/Office of Management and Budget (NIH/OMB) categories. Although few papers explicitly reported using the NIH/OMB categorization, a selection of these categories was often used in papers as well, whereas other categorizations were used very infrequently—one trial conducted across Asia, Europe, North America, and South America reported numbers for “Caucasian,” “African,” “Hispanic,” “East Asian,” and “West Asian” participants, and a paper about a trial conducted in the UK and Singapore reported the numbers of “Afro-Caribbean,” “Asian,” and “Caucasian” participants.

The median reported percentage of White participants in all studies was 94.7% (interquartile range [IQR]: 81.0–96.7%). This percentage of White participants was invariably high across both trials that did and those that did not use specific eligibility criteria (see Table S2 in supporting information). Only seven studies reported the number of participants with a Latinx (Latina/o) ethnic background (median: 5.6%, IQR: 4.2–11.4%); specifically, 20.0% of the trials that included a North American site for which race/ethnicity data was available (7/35) reported the number of participants with a Latinx ethnic background. Data regarding (non-)Latinx background was often presented separate from the number of participants in each racial group; it was therefore unclear how many participants with a Latinx background were included across racial groups (e.g., Latinx–White). The median percentage of Black/African American participants was 1.2% (IQR: 0.4–1.7%), and the median percentage of Asian participants was 4.4% (IQR: 0.3–17.3%; NB: three studies from Asia had samples consisting of 100% Asian participants). The median percent of other or multiracial participants was 0.9% (IQR: 0.0–1.9%).

We found no statistically significant relationship between the percentage of White participants and the study start year (*ρ* = –.26, *P* = .09). Of the studies for which a published paper was available 47.2% (25/53) reported the number of people who did not meet the eligibility criteria. Only 17.0% (9/53) specified which criteria most frequently were the cause of participant exclusion. Although one study (NCT00105547) reported whether the excluded and included patients differed on age and sex, none of the studies reported whether included and excluded participants differed on race/ethnicity.

Of the studies reporting race/ethnicity, none explicitly referred to socioeconomic status (SES), while 41.3% (19/46) reported on the participants’ education level. We extracted the mean education level of the total sample for each of these studies and calculated the average of the reported means across placebo and intervention groups for studies that did not report the total sample mean. The average mean number of years of education across these studies was 13.3 years, and a higher mean level of education was significantly correlated with a higher percentage of White participants included in the trial (*ρ* = .61, *P* = .02).

### 3.2 | Eligibility criteria

#### 3.2.1 | Criteria related to medical conditions

The frequency of exclusion criteria related to medical conditions is displayed in the first columns of Table 1, ranked from most prevalent (top) to least prevalent (bottom). In the remaining columns to the right, we present the prevalence of these medical conditions in several ethnoracial groups to provide context for the potential impact on ethnoracial diversity of participants. In addition to ethnoracial groups within the United States^19^ (non-Latinx White, Latinx, non-Latinx Black, American Indian/Alaska Native), we have included prevalence estimates from the Indigenous Australian population^20^ as an example to illustrate the potential impact of eligibility criteria on an international scale (see note to Table 1 for additional sources used to compile this table).

Non-AD neurological diseases and (major) psychiatric disorders were used as an exclusion criterion in more than three quarters of the included AD trials (Table 1, column 3), followed by cardiovascular disease (71.3%) and a history of cerebrovascular disease (68.3%). The last five columns of Table 1 demonstrate that the prevalence of some medical conditions is higher in either non-Latinx Black US residents, Latinx US residents, American Indian/Native Alaskan US residents, or Indigenous Australians than in non-Latinx White US residents or non-Indigenous Australians: diabetes, major psychiatric disease, cerebrovascular disease, renal disease, alcohol/substance use disorder, liver disease, higher weight/body mass index (BMI), and human immunodeficiencey virus (HIV) diagnosis rates. For diabetes, studies sometimes referred to specific HbA1c levels, but these levels varied substantially from <6.0% to <9.0%; other studies included “insulin dependent” diabetes, “poorly controlled” diabetes, or merely “diabetes.” Studies with a BMI criterion mostly required participants to have a minimum BMI of 18 or higher, but the upper cut-off value varied considerably from 28 to 40. Weight criteria specified a minimum weight of between 35 and 45 kg (≈77–99 pounds), mostly with a maximum of 120 kg (≈265 pounds). For hepatic disease, specific alanine transaminase (ALT; 1.5–3 times upper limit of normal, or ULN), aspartate transaminase (AST; 1.5–3 times ULN), and/or bilirubin (1.5–2.5 times ULN) cut-off levels were generally defined. For renal conditions, some studies referred to specific levels of creatinine clearance, whereas others only described “severe” renal disease, “impaired renal function,” or specified dialysis requirement as the exclusion criterion.

#### 3.2.2 | Criteria related to study procedures

Caregiver attendance was the most prevalent criterion related to study procedures (80.2%, see Table 2), which often specified that the same caregiver had to attend all study visits and sometimes that the caregiver either had to live at the patient’s home or had to visit a minimum number of times (range: <1–5 times/week) or hours per week (range: 4–24 hours/week). Some studies were more flexible, for example, by requiring the caregiver to accompany the patient only on key followup visits and allowing the patient to be accompanied by a “delegate” on the other visits. Written informed consent (52.5%) and a contraindication to undergoing positron emission tomography (PET)/magnetic resonance imaging (MRI; 51.5%) were used as a criterion in the majority of the included AD clinical trials.

Of the 19 studies using an education criterion, eight studies also allowed a work history consistent with no intellectual disabilities. For language fluency, most studies required fluency in the test language (n = 11), in the “local” language (n = 11), or in English (n = 8), while four studies allowed fluency in one of a number of languages. One study allowed fluency in any language with sponsor approval, as long as (1) staff were also fluent in that language, and (2) required study documents were available in that language. A subset of studies (14.9%) included a criterion whether patients or patient–caregiver dyads were likely to complete the study in the opinion of the investigator; an operationalization of this criterion was not provided.

#### 3.2.3 | Criteria related to diagnostic tests and questionnaires

Cognitive tests, batteries, or screeners were used as an inclusion criterion in nearly all studies, with little variety in the tests that were used; the MMSE score was a criterion in over 90% of the studies (Table 3). Aside from the MMSE, a handful of other screening tests/short batteries were used, such as the Repeatable Battery for the Assessment of Neuropsychological Status (RBANS^21^), the Alzheimer’s Disease Assessment Scale-Cognitive Subscale (ADAS-Cog^22^), and the Montreal Cognitive Assessment (MoCA^23^). Additionally, some studies used memory-specific tests: the Free and Cued Selective Reminding Test (FCSRT^24^), tests from the Wechsler Memory Scale–Revised (WMS-R^25^), and the International Shopping List Test (ISLT^26^). One study used different cut-off scores for the test they used (WMS-R) to correct for education (0–7, 8–15, and ≥16 years); none of the other studies described different cut-offs based on demographic or sociocultural characteristics known to impact cognitive test performance (e.g., age, sex, ethnicity, quality of education, acculturation, etc.).

In addition to cognitive tests, roughly one-third of the trials used the Clinical Dementia Rating (CDR^27^) global score as a criterion. A similar proportion of studies used a measure of psychiatric symptoms as part of the eligibility criteria. For depression, the 15-item version of the Geriatric Depression Scale (GDS^28^) was used most often, as well as the Hamilton Depression Rating Scale.^29^ The allowed range of scores for the GDS was relatively homogeneous across studies: the majority of studies (n = 22, 88% of studies with GDS) included patients with a score below 6 or 7, one study used the original 30-item version and used a cut-off score of ≤10, and two studies using a cut-off of <8 did not specify whether the long or short version of the GDS was used. The Columbia Suicide Severity Rating Scale^30^ was used a few times, but the majority of studies with a suicide risk criterion left the interpretation of this criterion to the opinion of the investigator (in contrast with depressive and cognitive symptoms).

##### Diagnostic tests and screeners: the use of the MMSE, CDR, and GDS over time

**FIGURE 2:**
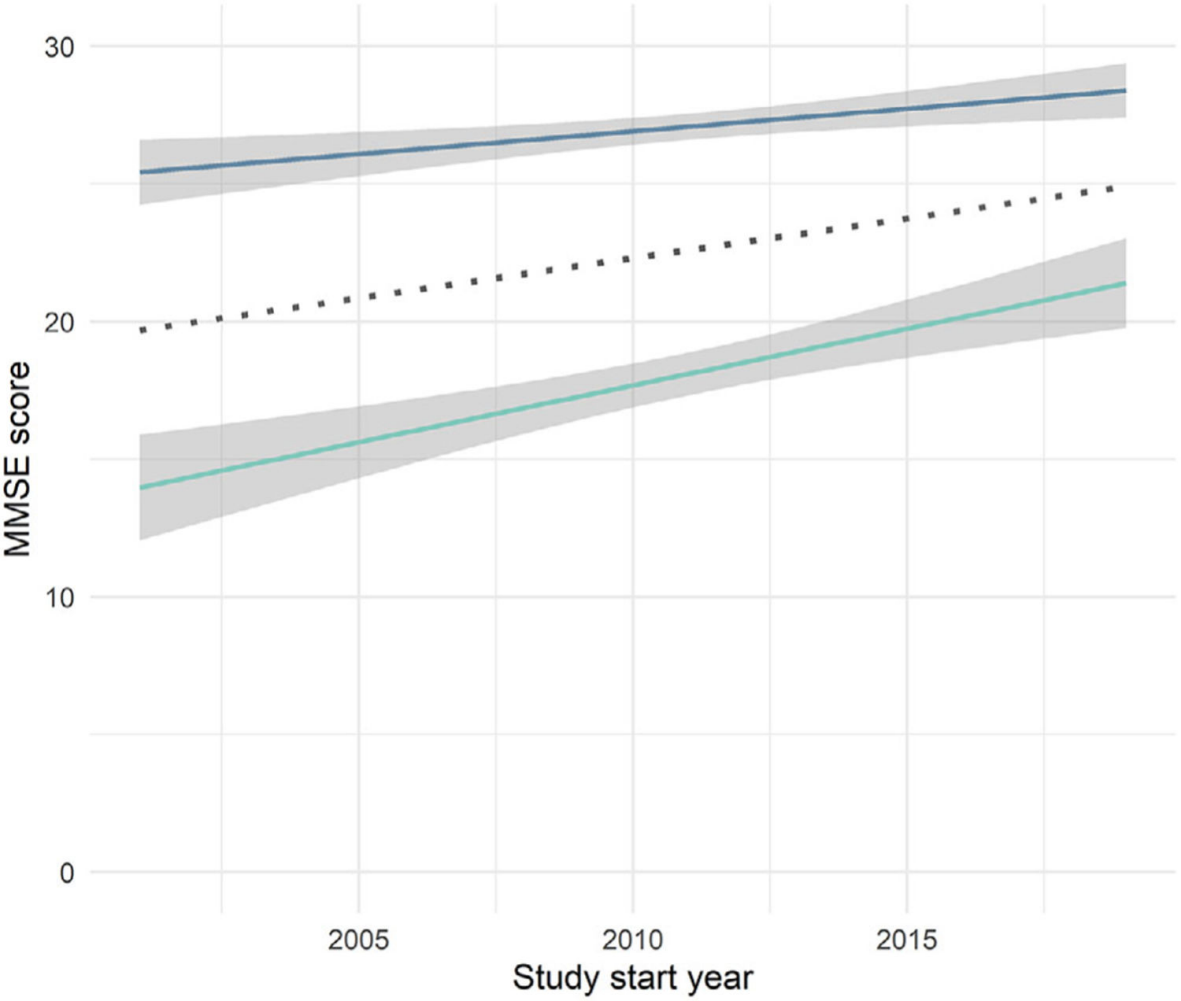
Changes in Mini-Mental State Examination (MMSE) upper and lower cut-off scores (midpoint in dotted line)

Additional Cochran-Armitage trend analyses of the use of the MMSE revealed that the study start year did not differ between studies with or without an MMSE-eligibility criterion (Z = 0.14, *P* = .89); that is, the MMSE cut-off scores were not used significantly less (or more) often with time. As displayed in Figure 2, the cut-off score for the MMSE increased over time (MMSE lower limit *ρ* = 0.53, *P* < .001; MMSE upper limit *ρ* = 0.48, *P* < .001). Furthermore, the range of allowed MMSE scores narrowed over time (*ρ* = –0.44, *P* < .001). Similar to the MMSE, the Cochran-Armitage trend test showed that there was no statistically significant increase or decrease in the use of the GDS by study year (Z= 0.0, *P* = .99); the CDR, however, was used significantly more frequently in later years (Z = –2.48, *P* = .01).

## 4 | DISCUSSION

In this systematic review, we aimed to (1) investigate the level of participant diversity in AD clinical trials targeting A*β* and tau; (2) identify which eligibility criteria have been used and how these criteria were defined; and (3) discover whether the use of criteria related to cognitive and neuropsychiatric instruments changed over time. The results showed that study samples were predominantly composed of White individuals, and ethnoracial diversity levels did not show a significant increase (or decrease) over time. Some of the most frequently reported criteria were the exclusion of participants with non-AD neurological disease, psychiatric illness, cardiovascular and cerebrovascular disease, obligated caregiver attendance, and cognitive impairment as defined by a specific score on the MMSE. The MMSE was used in an overwhelming majority of cases as the main cognitive eligibility criterion and was used consistently over time, with cut-off scores increasing over the years, but with the range of allowed scores decreasing over the years. The criteria related to medical conditions and study procedures often were not well operationalized and cut-off scores were often wide ranging. In addition to these main aims, our goal was to discuss how these eligibility criteria may have affected diversity levels. In the following paragraphs, we will discuss the main outcomes of this review and provide recommendations for future clinical trials, an overview of which can be found in Table 4.

We could not retrieve race/ethnicity data for more than half of the studies included in this review; for those studies for which a paper was published, a little over three quarters reported race/ethnicity data. This is somewhat higher than in a review of cholinesterase inhibitors and memantine randomized controlled trials (59.2%^5^). The studies that reported race/ethnicity data included an overwhelming majority of White participants (≈95%), and no significant increase or decrease in this ratio was observed over time. For most trials, data regarding Latinx ethnicity was not reported, and in the handful of cases in which it was described, it was presented separately from the numbers by racial group. It was therefore not possible to determine how many Latinx versus non-Latinx participants were included, and whether these proportions may have changed over time. However, based on the studies that did report the number of Latinx participants, as well as the data from Black, Asian, and other racial groups, it seems unlikely that Latinx participants were well represented. This lack of diversity, as well as the underreporting of Latinx background are particularly notable for studies with a North American site (79.2%), given the rapidly increasing diversification of the United States during this review period. Whitfield et al.^31^ describe how, as the ratio of White participants to other ethnoracial groups increases, the statistical power to detect group differences decreases drastically, and samples will typically have to include a larger proportion of diverse ethnoracial participants than a representative sample of the general population (e.g., more than 15% Black participants in the sample). As it stands, the limited percentage of ethnoracially diverse individuals precludes sufficiently powered analyses of safety and efficacy across ethnoracial groups. In addition, currently used racial/ethnic categories themselves may need to be revised to fully represent global diversity—for example, categorizing all individuals from Europe, North Africa, and the Middle East as “White” does not do justice to the diversity within and between persons originating from these regions.

Our results showed that trials targeting A*β* or tau in AD often provide unclear definitions of their eligibility criteria; these imprecise definitions, such as “diabetes” or “impaired renal function” (not further specified), likely result in the exclusion of all or most patients with a specific medical condition. When specific ranges on indices of certain medical conditions were provided, such as BMI or ALT/AST levels, the allowed ranges differed substantially between studies. There thus seems to be a lack of consensus on how these conditions are best defined in the context of A*β* and tau trials. These ill-defined eligibility criteria may particularly affect the inclusion of underrepresented populations that are characterized by health disparities. Kim et al.^32^ made several suggestions to broaden inclusion criteria in oncology trials that may provide inspiration for AD trials. One of these recommendations is to include persons living with HIV (PWH) based on current and past CD4+ and T-cell counts instead of excluding all PWH—unless antiretroviral therapy is expected to interact with the investigational product. Additionally, one might take into consideration whether PWH are medically stable and whether they have a (non-)detectable viral load. Furthermore, Kim et al.^32^ provided examples of how to improve the clarity of the definitions used in clinical trials eligibility criteria, such as the use of validated clinical classifications (of disease staging) as opposed to more generic definitions.

With regard to the impact of criteria related to medical conditions on the inclusion of ethnoracially diverse groups specifically, it is still uncertain if, how, and when race corrections should be used to evaluate various clinical laboratory results as indicators of specific medical conditions, such as indicators of kidney functioning^33^ and several other common laboratory values.^34^ Although such race corrections could potentially make the process of inclusion in clinical trials more inclusive, they may also inadvertently perpetuate or amplify existing disparities.^35^ The field is in need of expert guidance to reach a consensus on whether and when to apply these race corrections.

Criteria related to undergoing study procedures were commonly part of the eligibility criteria. In the following paragraphs, the eligibility criteria related to language, education, caregiver attendance, written informed consent/reading and writing abilities, and whether patients are considered likely to complete the study, are discussed in more detail, specifically in the context of the inclusion of diverse individuals.

First, language requirements, such as fluency in the English language, were included in more than one third of the clinical trials. Depending on their definition, specific language requirements may lead to disproportionate exclusion of individuals from underrepresented populations. The lack of guidance on how to handle language barriers in clinical trials was acknowledged as a problem by multicenter research ethics committees in the UK.^36^ A more inclusive solution may be to allow fluency in any preferred language, as long as the required test materials are available in that language and there is a staff member available who speaks the language to the degree necessary for cognitive testing—as was allowed in one trial (NCT00676143). This would, however, require the development/adaptation and validation of test materials across a number of languages. In addition, it may be worthwhile to investigate if assessment with experienced formal interpreters could be a viable option at study sites where the population is exceptionally diverse.

Regarding education, a minimum of 6 years of formal education was often used as a criterion—sometimes stating this was to ensure that patients with intellectual disabilities were not included. This criterion is problematic for several reasons; first, many diverse elderly patients across the world did not receive any formal education during childhood due to reasons other than intellectual disabilities—such as a lack of financial means or a large geographic distance to educational facilities (e.g., in first generation immigrants in Europe). Second, mandatory primary education across the world has historically been variable— although some countries required 6 years of primary education, others may have required only 4 or 5. Therefore, years or level of education cannot serve as a suitable proxy for intellectual disabilities in diverse patients. Some studies acknowledged thesebarriers by allowing people with a work history consistent with no intellectual disabilities to participate in the study. Future studies should focus on developing ways to screen for intellectual disabilities that do not result in the exclusion of patients without intellectual disabilities who had limited access to formal education.

Several studies included a criterion that patients should be likely to complete the study. However, the interpretation of this criterion often was not defined, requiring the investigator to make this judgment call. Although such a criterion may be necessary to prevent costly missed visits in clinical trials, especially for studies using PET-ligands, likeliness to complete should be well defined at the outset. For example, a protocol may state that the patient and caregiver should complete a first run-in period of a specific number of screening visits fully compliant with the specified study procedures and in line with a specified time schedule. If this criterion is left undefined, it may prove problematic, as studies have indicated that participant selection may be influenced by implicit bias of the clinicians, that is, compliance stereotyping.^37^

More than three quarters of the studies required some form of caregiver participation, often explicitly stating caregivers had to engage in frequent contact with patients—one study required caregivers to spend at least 24 hours per week with the patient. In some diverse ethnoracial groups, the main caregiver is often an adult child, rather than a spouse,^38–40^ and previous research has indicated that adult children are less likely than spouses to be eligible to participate alongside patients in dementia clinical trials.^41^ Adult–child caregivers are more likely to still be active in the workforce,^39^ potentially limiting their opportunities to engage in frequent study visits due to the practical and financial burden of missed work. Researchers may provide more flexibility by allowing others to accompany patients on a subset of visits; by having appointments taking place outside of weekday business hours; or by exploring options for remote administration of interviews, such as over the phone or via video calls.^39^

More than half of the AD clinical trials in this review explicitly required written informed consent. Although this currently seems to be the standard, requiring written informed consent will lead to the exclusion of people with low literacy skills—either because these patients will not be asked, or because they will be hesitant to sign a document they have difficulty understanding. Globally, ≈781 million adults are illiterate, with a high prevalence in lower- and middle-income countries,^42^ although disparities in literacy are also prevalent in some underrepresented populations in high-income countries. For example, so-called “guest workers” in Europe often received little if any formal education,^43,44^ and Latinx adults—and to a lesser degree Black and American Indian/Alaska Native adults—in the United States were overrepresented in the “below basic” level on the National Assessment of Adult Literacy.^45^ To facilitate the enrollment of underrepresented populations, informed consent procedures will have to be tailored to patients and caregivers with low literacy skills. Over two decades ago, the US Food and Drug Administration (FDA) described the possibility of non-written consent procedures in illiterate English-speaking subjects, in which an impartial third party cosigns the consent document, preferably with a videotape recording.^46^ A recent study in a different medical field (cardiology/endocrinology) has indicated that using a video informed consent procedure can increase the enrollment of patients from underrepresented populations.^47^ As an additional example, in India, audiovisual recording of the informed consent procedure has been mandatory since 2013, and standard operating procedures have consequently been developed.^48^ AD research would benefit from efforts to incorporate alternatives to written informed consent developed in other research areas that include diverse and vulnerable populations, as well as from initiatives examining the feasibility of integrating such approaches in AD research.

Regarding cognitive screening tests and questionnaires, we found that the MMSE was used almost invariably as an inclusion criterion, and its use remained stable over time, with cut-off scores even increasing over the years. This is notable, given the fact that Schneider et al.^14^ warned about the use of the MMSE in dementia trials in 1997. There is an abundant literature describing how MMSE-scores are substantially influenced by literacy and education^49–51^ and likely also by cultural background.^50^ In particular the subtests of orientation to time and place, serial 7s, figure copy, writing, and reading will be substantially influenced by someone’s educational and cultural background.^52^ Developing alternatives to written informed consent will only solve half of the problem as long as the cognitive tests used for screening and to measure primary and secondary outcomes require reading and writing skills. Moving forward toward more valid and inclusive global clinical trials will entail using other cognitive tests that are more suitable for diverse populations. For instance, the Rowland Universal Dementia Assessment Scale (RUDAS^53^)—a test to assess the general level of cognitive impairment—or the International Shopping List Test^26^—for the inclusion of patients with memory impairment specifically—may be relevant options for further study. Before any instrument is selected for a clinical trial, it is imperative that a thorough review of the literature is carried out to determine whether the instrument is a valid and reliable measure of cognition in all groups that are to be included in the trial. As selection bias is often present in reliability/validity studies— for example, by excluding persons with low education levels or limited language fluency—it may be necessary to specifically check the demographic characteristics of these original study samples to ensure they reflect the intended trial sample. At a minimum, trials can be made more equitable by using different cut-off scores for groups with different levels of education in cognitive screeners and memory tests, as was done by one trial in this review (NCT00890890).

In addition to the MMSE, this study showed a rise in the use of the CDR as an inclusion criterion. The CDR has considerable merits, but researchers and clinicians need to be aware of possible cultural differences that may bias the results, such as (1) downplaying of cognitive symptoms out of respect for older family members, (2) different perceptions of what “normal” daily functioning may entail, (3) the need for adaptations to questions relating to hobbies that may be uncommon in some groups—for example, crossword puzzles—and social or cultural practices, (4) thepoten tialinflu en ceoftraditi onalge nderroles, and (5) the potential influence of limited literacy on some activities of daily life.^54^ Aside from the extensive training that is already needed to administer the CDR in a reliable and valid way in the general population, it is likely that additional training and/or adaptations to the instrument itself are needed to make it more suitable for the assessment of diverse populations across the globe.

In addition to these specific recommendations pertaining to criteria related to medical conditions, undergoing study procedures, and cognitive screeners and questionnaires, some general recommendations may further improve inclusion of underserved populations in AD clinicaltrials. In the design phase, the FDA^55^ specifically recommends revisiting and revising the criteria when moving from a restrictive phase II to a more inclusive phase III trial.^32,55^ Furthermore, they encourage the inclusion of samples known as “expansion cohorts” in trials—consisting of patients with specific comorbidities that may not fit the inclusion criteria for the main study—to determine the safety of doses in these populations as well.^32^ Aside from changes to the trial design, more insight can be gained into the mechanisms behind the underrepresentation of diverse patients in clinical trials, if studies were to report the ethnoracial characteristics of all patients that (1) were considered for eligibility, (2) were invited, (3) were screened, and (4) were excluded/screen failed. In addition, reports should provide specifications regarding the eligibility criteria that were most often the reason for exclusion.

Although not technically part of the CONSORT (Consolidated Standards of Reporting Trials) guidelines,^56^ a short summary of the main reasons for exclusion may provide valuable insights to researchers on the eligibility criteria that have the strongest effect on eligibility. This information was only provided in a handful of studies in this review, and none of the studies specified whether there was a disproportionate exclusion of patients from underrepresented populations. It therefore remains unclear whether there was a disproportionate exclusion of patients from these groups based on overly strict eligibility criteria, or whether these patients were not invited in the first place or did not consent to study participation after invitation. For example, patients from underrepresented populations may experience geographical, financial, or logistical barriers that prevent them from participating in research.^55,57^ Additionally, recruitment strategies need to be tailored to suit the needs of underrepresented populations, such as by investing in community-outreach programs, trust-building initiatives, and cultural-sensitivity training.^10,36,58,59^ Financial support from funding agencies and/or the trial sponsor to facilitate such initiatives may be needed. In addition, more general financial or regulatory incentives from funding organizations or governmental bodies to actively enroll patients from underrepresented populations may further improve inclusion, for example, similar to the changes in the field of pediatrics, in which the Pediatric Research Equity Act (PREA) now requires manufacturers to complete studies in children if a substantial number of children is expected to use the drug.^60^

Although this review specifically examined race/ethnicity, we acknowledge that race is a social construct and that health disparities are often driven by social determinants of health, such as education, literacy, socioeconomic status, racially patterned social stress, and access to care.^61–63^ Although some trials in this review with race/ethnicity data reported the education level of the included participants, none mentioned SES. This limited reporting of social determinants of health is in line with a previous review in symptomatic treatment of AD, in which no studies reported on variables such as lifetime occupation, individual/household income, or wealth, and few studies on education.^64^ It remains unclear how these variables may have affected enrollment of diverse participants in the trials included in this review; however, participants are often recruited in memory clinics, and these facilities may not be accessible to some underrepresented groups, for example because of limited health literacy,^65^ or because medical care is expensive and insufficiently covered by insurance.^66^

Several limitations to this review should be mentioned. Although we did not exclude studies based on the language in which the record was written, our study did not identify any articles that were not written in English. Therefore, some local trials may have been missed. Second, race/ethnicity data was not available for a substantial number of studies, and the full protocols describing all eligibility criteria were only available for about one fifth of the included trials. As can be seen in the supporting information, the frequencies of the eligibility criteria may differ between studies with and without a full protocol available, and the rates we presented in this review may be an underestimation of the actual frequencies. For example, it seems unlikely that only slightly more than half of the clinical trials required written informed consent, particularly as the studies without such a criterion did not describe any alternative consent requirements. Likewise, trials that did not report race/ethnicity data may have included even fewer diverse participants—or, less likely, more—than the studies that did report race/ethnicity data. Third, in this review, we presented data from diverse ethnoracial populations in Australia and the United States alongside the frequencies of the eligibility criteria related to medical conditions to provide the reader with a better sense of the potential impact on diversity in clinical trials. These populations cannot be seen as directly representative of all underrepresented populations across the world, and given that these data were obtained in the general population, health disparities may actually be even more systemic and striking when zooming in on elderly populations specifically. For example, the prevalence of overweight and obesity in indigenous populations in Australia is 35% in those aged 15 to 17, but rises to 80% in those 55 and over.^20^ Although we only showed data from the United States and Australia, similar health disparities are observed in populations outside those two countries, such as across different ethnoracial groups in Europe—particularly in the prevalence of diabetes, stroke, hypertension, and cardiovascular disease,^67–69^ but also in kidney disease.^70,71^ Fourth, it is important to note that the data based on Latinx American samples is based on a pan-Latinx construction of this population. These studies did not account for the significant within-group variance that has important implications for health disparities and cognitive test performance (e.g., origin/nativity [Mexican, Puerto Rican, etc.], acculturation). Fifth, we only focused on A*β* and tau trials in this review. Although many of these recommendations can likely also be applied to other types of trials across neurodegenerative diseases, such as lifestyle trials like World-Wide FINGERS,^72^ some of these trials will come with their own unique challenges—such as a lack of suitable cross-cultural instruments measuring social cognition, language, and behavioral changes in frontotemporal dementia trials^73^ as well as issues regarding the applicability of the diagnostic criteria for primary progressive aphasia subtypes across global languages, such as Chinese.^74^ Last, we were unable to determine the direct effect of each criterion on the representation of diverse individuals using inferential statistics. Several factors precluded such analyses, such as the fact that some criteria were used either very infrequently or invariably (e.g., the MMSE, Table S2), as well as the fact that race/ethnicity data was not reported for each global region/country specifically, precluding any comparisons of the makeup of the study samples with a priori disease estimates in the general populations in these countries/regions. The contribution of each individual eligibility criterion to the underrepresentation of diverse individuals across trials therefore remains unclear—even more so given the underreporting of the main reasons for exclusion.

Both federal law (Public Health Service Act §492B^75^) and NIH policy^76^ require studies involving human subjects to address the inclusion of “minorities,” and Alzheimer Europe^77^ similarly calls upon researchers, ethics committees, and funders to address inequity in research. This review illustrates that there is a continuous, systemic underrepresentation of ethnoracially diverse groups in AD clinical trials.To generalize safety and efficacy data of AD clinical trials to the general population, more diverse individuals need to be enrolled, and modifying or changing the eligibility criteria in AD clinical trials may play a key role in reaching this goal.

## Supplementary Material

Supporting Information

## Figures and Tables

**TABLE 1:** Frequencies of eligibility criteria related to medical conditions and prevalence of medical conditions in American and Australian ethnoracial groups*

	Criterion frequency in all trials (N = 101)		% in n-L White Americans	% in Latinx Americans	% in n-L Black Americans	% in American Indian and Alaska Native	% in Indigenous Australians^†^
Other neurological disease	81	80.2%	—	—	—	—	—
Psychiatric disorder	79	78.2%	6.9%	9.4%	9.7%	—	12% (9.6%)
Cardiovascular disease	72	71.3%	11.5%	8.2%	10.0%	14.6%	13% (1.2x)
Cerebrovascular disease	69	68.3%	2.6%	2.5%	3.9%	3.0%	—
- Hachinski ischemia scale score >4	53	52.5%	—	—	—	—	—
- Cerebrovascular evidence on MRI	48	47.5%	—	—	—	—	—
Childbearing/conception	62	61.4%	—	—	—	—	—
Unspecified systemic illness	62	61.4%	—	—	—	—	—
Alcohol or drug abuse	59	58.4%	8.4%	8.6%	7.4%	14.9%	18% (19%)
Vitals or lab abnormalities	53	52.5%	—	—	—	—	—
Infections/infectious diseases	50	49.5%	—	—	—	—	—
- HIV status^‡^	26	25.7%	4.8^‡^	16.4^‡^	39.2^‡^	7.7^‡^	5.5^‡^ (4.5^‡^)
Liver disease	48	47.5%	1.7%	2.7%	1.1%	2.5%	15%–23% (1.4x–2.1x)
Autoimmune disease	47	46.5%	22.0%	16.8%	21.0%	30.6%	10.0% (1.1x)
Renal disease	46	45.5%	2.0%	2.2%	3.1%	—	3.0% (≈3.7x)
Seizure disorder	44	43.6%	—	—	—	—	—
Cancer	41	40.6%	9.1%	4.2%	5.1%	7.1%	1.7% (1.5%)
Respiratory illness^§^	26	25.7%	7.5%^§^; 3.6%	6.0%^§^; 2.7%	9.1%^§^; 3.4%	9.5%; —	18% (1.9x) —
Endocrine dysfunction	25	24.8%	—	—	—	—	—
Brain/head trauma	25	24.8%	—	—	—	—	—
Diabetes^¶^	23	22.8%	8.6%^¶^; 13.0%	13.2%^¶^; 21.5%	13.1%^¶^; 19.6%	23.5% —	11% (3.3x) —
Weight or BMI cut-off	21	20.8%	31.0%	34.9%	38.0%	48.1%	37% (1.6x)
Gastrointestinal disease	18	17.8%	5.7%	4.3%	4.9%	8.3%	—
Excessive smoking (≥20 cigarettes per day)	9	8.9%	—	—	—	—	—
CNS inflammation	8	7.9%	—	—	—	—	—
Systemic inflammation	6	5.9%	—	—	—	—	—

Abbreviations: BMI, body mass index; CNS, central nervous system; HIV, human immunodeficiency virus; MR, magnetic resonance imaging; n-L, non-Latinx.

* 2018 US National Health Interview study data^19^ and 2015 Australian Institute of Health and Welfare data are presented^20^ (unless otherwise specified), providing prevalence rates for the following specific conditions within the broader categories specified in the first column: psychiatric disorders = moderate to severe depressive symptoms (US^78^) versus feeling depressed (AUS); cardiovascular disease = any; cerebrovascular disease = stroke; alcohol or drug abuse = substance dependence or abuse (US^79^) versus lifetime risky alcohol consumption (AUS); infections – HIV status (US^80^); autoimmune disease = arthritis diagnosis; renal disease = weak or failing kidneys (USA) versus chronic kidney disease stages 3–5 (AUS); liver disease = any (US) versus abnormal ALT/GGT (AUS); cancer = any; weight or BMI = obesity; gastrointestinal disease = ulcers (duodenal, stomach, peptic).

† In parentheses: times increased risk compared to non-Indigenous Australians or prevalence rate in non-Indigenous Australians.

‡ Diagnosis rate per 100,000.

§ Respiratory illness = current asthma (top) and chronic bronchitis (bottom).

¶ Diabetes = diagnosed (top) versus diagnosed and undiagnosed combined (bottom^81^).

**TABLE 2:** Frequencies of criteria related to undergoing study procedures

	Criterion frequency in all trials (N = 101)	
Caregiver attendance	81	80.2%
Written informed consent	53	52.5%
Contraindication to MRI/PET	52	51.5%
Adequate sensory abilities	42	41.6%
Language ability	35	34.7%
Residence in the community	35	34.7%
Caregiver consent	28	27.7%
Education requirement	19	18.8%
Reading or writing ability	19	18.8%
Determined likely to complete	15	14.9%
Recent hospitalization	4	4.0%

Abbreviations: MRI, magnetic resonance imaging; PET, positron emission tomography.

**TABLE 3:** Frequencies of neurocognitive and neuropsychiatric screening tests and measures

	Criterion frequency in all trials (N = 101)	
**COGNITIVE TESTS**		
MMSE	91	90.1%
Memory-specific test*	7	6.9%
RBANS	4	4.0%
ADAS-Cog	3	3.0%
MoCA	1	1.0%
**GLOBAL & FUNCTIONAL MEASURES**		
CDR	36	35.6%
Eastern Cooperative Oncology Group status	1	1.0%
FAQ	1	1.0%
**PSYCHIATRIC ASSESSMENTS**		
Geriatric Depression Scale	25	24.8%
Hamilton Depression Rating Scale	6	5.9%
Other depression instrument	1	1.0%
C-SSRS	5	5.0%
Other/unspecified suicide or self-harm risk scale	14	13.9%

Abbreviations: ADAS-Cog, Alzheimer’s Disease Assessment Scale-Cognitive Subscale; C-SSRS, Columbia Suicide Severity Rating Scale; CDR, Clinical Dementia Rating; FAQ, Functional Activities Questionnaire; MMSE, Mini-Mental State Examination; MoCA, Montreal Cognitive Assessment; RBANS, Repeatable Battery for the Assessment of Neuropsychological Status.

* Includes Free and Cued Selective Reminding Test (FCSRT), Wechsler Memory Scale-Revised (WMS-R), and International Shopping List Test (ISLT).

**TABLE 4:** Issues with eligibility criteria of clinical trials and recommendations

Issue/criterion	Recommendations
**Overarching issues**	
-Race and ethnicity often were not reported	-Improve reporting
-Current race/ethnicity definitions not globally suitable	-Critically examine and improve definitions of race/ethnicity
-It is unclear how many diverse patients are invited, screened, and excluded	-Improve reporting
-It is unclear which criteria lead to exclusion	-Improve reporting
-Criteria from phase II copied to and expanded on in phase III	-Revisit/revise all criteria in moving from phase II to phase III
**Criteria related to medical conditions**	
-Imprecise/unspecific definitions of medical conditions	-Use validated, internationally recognized clinical classifications (of disease staging)
-Variation in cut-offs for specific medical conditions	-Organize expert consensus meetings to determine appropriate cut-offs in AD research
-It is unclear if race corrections should be used or not	-Organize expert consensus meetings to determine whether and when to apply race corrections
-Exclusion of all patients with a medical condition regardless of past/present health status	-Include more patients who can safely participate, for example, persons living with HIV who are medically stable and have a non-detectable viral load
-Questionable safety of drugs for patients with medical conditions due to exclusion	-Use expansion cohorts to study safety
**Criteria related to study procedures**	
-Language fluency as a barrier to participation	-Allow fluency in any language if adapted materials and staff speaking that language are available
-Lower educated individuals often excluded	-Allow persons with a work history consistent with no intellectual disabilities (ID) to participate
	-Investigate other ways to screen for ID
-Risk of compliance stereotyping if “likely to complete” is not defined	-Define “likely to complete” before trial
-Caregiver attendance as a barrier to participation	-Allow others to accompany patient on subset of visits
	-Plan appointments outside business hours
	-Explore remote interviewing options
-Written informed consent as a barrier in persons with limited literacy/education	-Explore alternatives for written informed consent, such as video informed consent
**Criteria related to neurocognitive and neuropsychiatric measures**	
-MMSE is unsuitable for diverse populations	-Consider alternative, more widely applicable tests
	-Use different MMSE cut-offs depending on education and other relevant variables
-CDR may be biased due to cultural differences	-Consider adaptations to the instrument/questions
	-Provide additional training to staff

Abbreviations: AD, Alzheimer’s disease; CDR, Clinical Dementia Rating; MMSE, Mini-Mental State Examination.

## RESEARCH _IN CONTEXT_

1. **Systematic review**: We reviewed the published literature and clinical trial registries to examine participant diversity in Alzheimer’s disease drug trials, as well as developments in eligibility criteria for these trials. The trials covered a time period from 2001–2019.
2. **Interpretation**: Study samples in AD trials were predominantly white, and participant diversity did not change overtime. Several characteristics of eligibility criteria may lead to systemic exclusion of diverse populations, such as the use of specific cognitive tests, requirements regarding language fluency, education, or caregiver attendance, and the exclusion of patients with comorbid conditions.
3. **Future directions**: This review highlights several eligibility criteria that require more research/consensus, such as how to best operationalize medical conditions and which cognitive and clinical tests may best be used in diverse populations. In addition, it highlights the need for revision of race/ethnicity categorization and for better reporting of diversity-related data.
