# Supplementary material for: Diversity in Alzheimer’s disease drug trials: The importance of eligibility criteria
Source: Alzheimers Dement. Author manuscript; Available in PMC 2023 Apr 1. (PMC8964823; doi:10.1002/alz.12433)
Supplement: Supporting Information [file NIHMS1775420-supplement-Supporting_Information.docx]

| **Supplementary Table 1. List of included trials and data sources** | | | | | | | | | | |
| --- | --- | --- | --- | --- | --- | --- | --- | --- | --- | --- |
|  | **Trial Registry** | | | **Trial Details** | | | | | **Additional Sources** | |
| **DMT** | NCT# | EudraCT | ANZCTR | Phase | Year | Status | Sample size | Region (number of countries)* | Protocol | Paper |
| AADvac1 | NCT02579252 | 2015-000630-30 |  | 2 | 2015 | Complete | 208 | Europe (8) |  | - |
| ABvac40 | NCT03461276 |  |  | 2 | 2018 | Recruiting | - | Europe (4) |  | - |
| Aducanumab | NCT02477800 | 2015-000966-72 |  | 3 | 2015 | Terminated | 1647 | Asia (3), Europe (8), North America (2), Oceania (1) |  | - |
|  | NCT02484547 | 2015-000967-15 |  | 3 | 2015 | Terminated | 1638 | Asia (1), Europe (10), North America (2) |  | - |
|  | NCT03639987 | 2018-002102-31 |  | 2 | 2018 | Terminated | 500 | Europe (2), North America (2), Oceania (1) |  | - |
| AFFITOPE AD02 | NCT01117818 | 2009-016504-22 |  | 2 | 2009 | Complete | 335 | Europe (6) |  | Schneeberger et al. 2015 |
| Amilomotide (CAD106) | NCT00733863 |  |  | 2 | 2008 | Complete | 58 | Europe (4) |  | Farlow et al. 2015 |
|  | NCT00795418 |  |  | 2 | 2008 | Complete | 31 | North America (1) |  | Farlow et al. 2015 |
|  | NCT01097096 | 2009-012394-35 |  | 2 | 2009 | Complete | 121 | Europe (8), North America (2) | Yes | Vandenberghe et al. 2017 |
| AN1792 (AIP 001) | NCT00021723 |  |  | 2 | 2001 | Complete | 372 | Europe (?), North America (1) |  | Gilman et al. 2005 |
| Atabecestat | NCT02260674 | 2014-002159-24 |  | 2 | 2014 | Complete | 114 | Europe (6) |  | - |
| Avagacestat | NCT00810147 | 2008-005929-11 |  | 2 | 2008 | Complete | 209 | Europe (3), North America (1) |  | Coric et al. 2012 |
|  | NCT00890890 | 2009-010067-16 |  | 2 | 2009 | Terminated | 263 | Europe (4), North America (2) | Yes | Coric et al. 2015 |
| Azeliragon | NCT00141661 |  |  | 2 | 2005 | Complete | 67 | North America (1) |  | - |
|  | NCT00566397 |  |  | 2 | 2007 | Complete | 402 | North America (1) |  | Galasko et al. 2014 |
|  | NCT02080364 |  |  | 2 | 2014 | Terminated | 880 | Africa (1), Europe (2), North America (2), Oceania (2) |  | - |
| Lecanemab | NCT01767311 | 2012-002843-11 |  | 2 | 2013 | Active | 800 | Asia (2), Europe (7), North America (2) |  | - |
|  | NCT03887455 | 2018-004739-58 |  | 3 | 2019 | Recruiting | - | Asia (2), Europe (6), North America (2) |  | - |
| Bapineuzumab |  | 2004-004120-12 |  | 2 | 2004 | Complete | 26 | Europe (2) |  | Rinne et al. 2010 |
|  | NCT00112073 |  |  | 2 | 2005 | Complete | 234 | North America (1) |  | Salloway et al. 2009 |
|  | NCT00174525 |  |  | 2 | 2005 | Unknown | - | North America (1) |  | - |
|  | NCT00574132 |  |  | 3 | 2007 | Complete | 1331 | Europe (2), North America (2) | Yes | Salloway et al. 2014 |
|  | NCT00575055 |  |  | 3 | 2007 | Complete | 1121 | North America (1) | Yes | Salloway et al. 2014 |
|  | NCT00676143 | 2007-005995-14 |  | 3 | 2008 | Terminated | 1100 | Africa (1), Asia (1), Europe (16), North America (2), Oceania (2)  South America (2) | Yes | Vandenberghe et al. 2016 |
|  | NCT00663026 |  |  | 2 | 2008 | Complete | 79 | North America (1) |  | - |
|  | NCT00667810 | 2007-005994-79 |  | 3 | 2008 | Terminated | 901 | Africa (1), Asia (2), Europe (16), North America (3), Oceania (2), South America (2) | Yes | Vandenberghe et al. 2016 |
|  | NCT01254773 |  |  | 2 | 2010 | Complete | 146 | North America (1) |  | Brody et al. 2016 |
| Bryostatin-1 | NCT02221947 |  |  | 2 | 2014 | Terminated | 9 | North America (1) |  | Nelson et al. 2017 |
|  |  |  |  | 2 | 2019 | Complete | 147 | North America (1) |  | Farlow et al. 2019 |
| CHF 5074 | NCT01602393 | 2010-024270-19 |  | 2 | 2011 | Complete | 51 | Europe (1)  North America (1) |  | - |
|  | NCT01723670 |  |  | 2 | 2012 | Withdrawn | 0 | North America (1) |  | - |
| Colostrinin |  |  |  | 2 | 2004 | Complete | 105 | Europe (1) |  | Bilkiewicz & Gaus, 2004 |
| Crenezumab | NCT01397578 |  |  | 2 | 2011 | Complete | 91 | Europe (2), North America (1) |  | Salloway et al. 2018 |
|  | NCT01343966 | 2010-021926-37 |  | 2 | 2011 | Complete | 448 | Europe (4), North America (2) |  | Cummings et al. 2018 |
|  | NCT02670083 |  |  | 3 | 2016 | Complete | 813 | Asia (3), Europe (21), North America (4), Oceania (1) | Yes | - |
|  | NCT03114657 | 2016-003288-20 |  | 3 | 2017 | Complete | 806 | Africa (1), Asia (5), Europe (16), North America (3), Oceania (1), South America (3) |  | - |
| Daratumumab | NCT04070378 |  |  | 2 | 2019 | Recruiting | 15 | North America (1) | Yes | - |
| Donanemab | NCT03367403 |  |  | 2 | 2017 | Active | 266 | North America (2) |  | - |
| Elenbecestat | NCT02322021 |  |  | 2 | 2014 | Active | 71 | North America (1) |  | - |
|  | NCT02956486 | 2016-003928-23 |  | 3 | 2016 | Active | 950 | Asia (2), Europe (12), North America (2), Oceania (1), South America (1) |  | - |
|  | NCT03036280 | 2016-004128-42 |  | 3 | 2017 | Terminated | 950 | Africa (1), Asia (5), Europe (13), North America (3), South America (1) |  | - |
| ELND005 | NCT00568776 |  |  | 2 | 2007 | Complete | 353 | North America (2) |  | Salloway et al. 2011 |
| Etanercept | NCT01068353 | 2009-013400-31 |  | 2 | 2010 | Complete | 41 | Europe (1) |  | - |
| EVP-0962 | NCT01661673 |  |  | 2 | 2012 | Complete | 52 | North America (1) |  | - |
| Gantenerumab | NCT01224106 | 2010-019895-66 |  | 3 | 2010 | Active | 799 | Asia (1), Europe (16), North America (3), Oceania (1), South America (3) |  | Ostrowitzki et al. 2017 |
|  | NCT02051608 | 2013-003390-95 |  | 3 | 2013 | Active | 389 | Asia (2), Europe (16), North America (2), Oceania (1), South America (1) |  | - |
|  | NCT03443973 | 2017-001365-24 |  | 3 | 2017 | Recruiting | 750 | Asia (3), Europe (11), North America (3), South America (2) |  | - |
|  | NCT03444870 | 2017-001364-38 |  | 3 | 2017 | Recruiting | 750 | Asia (3), Europe (7), North America (2), Oceania (1), South America (3) |  | - |
| Gosuranemab | NCT03352557 | 2017-002901-37 |  | 2 | 2017 | Active | 654 | Asia (1), Europe (6), North America (1), Oceania (1) |  | - |
| IVIg | NCT00299988 |  |  | 2 | 2006 | Terminated | 24 | North America (1) |  | - |
|  | NCT00812565 |  |  | 2 | 2008 | Complete | 58 | North America (1) |  | - |
|  | NCT00818662 |  |  | 3 | 2009 | Complete | 390 | North America (2) |  | Relkin et al. 2017 |
|  | NCT01300728 |  |  | 2 | 2011 | Active | 52 | North America (1) |  | - |
|  | NCT01524887 | 2011-000914-21 |  | 3 | 2012 | Terminated | 508 | Asia (1), Europe (4), North America (2), Oceania (1) |  | - |
|  | NCT03319810 |  |  | 2 | 2017 | Complete | 5 | North America (1) | Yes | - |
| Lanabecestat | NCT02245737 | 2014-002601-38 |  | 3 | 2014 | Terminated | 2218 | Asia (2), Europe (9), North America (3), Oceania (1) | Yes | - |
|  | NCT02783573 | 2015-005625-39 |  | 3 | 2015 | Terminated | 3800 | Asia (4), Europe (10), North America (3) | Yes | - |
| LMTM | NCT00515333 |  |  | 2 | 2007 | Complete | 323 | Asia (1), Europe (1) | Yes | Wischik et al. 2015 |
|  | NCT01689246 | 2012-002866-11 |  | 3 | 2012 | Complete | 891 | Asia (4), Europe (9), North America (2), Oceania (1) | Yes | Gauthier et al. 2016 |
|  | NCT01689233 | 2012-002847-28 |  | 3 | 2012 | Complete | 761 | Europe (9), North America (2), Oceania (1) | Yes | Wilcock et al. 2018 |
| LY2886721 | NCT01561430 | 2011-005217-37 |  | 2 | 2012 | Terminated | 70 | Asia (1), Europe (3), North America (1) |  | - |
| LY3202626 | NCT02791191 |  |  | 2 | 2016 | Terminated | 316 | Asia (1), North America (2), Oceania (1) |  | - |
| NPO31112 | NCT00948259 |  |  | 2 | 2009 | Complete | 30 | Europe (1) |  | - |
| PBT2 | NCT00471211 |  |  | 2 | 2007 | Complete | 78 | Europe (1), Oceania (1) |  | Lannfelt et al. 2008 |
|  |  |  | ACTRN12611001008910 | 2 | 2011 | Complete | 42 | Oceania (1) |  | Villemagne et al. 2017 |
| Ponezumab | NCT00722046 |  |  | 2 | 2008 | Complete | 194 | Asia (1), Europe (2), North America (2), Oceania (1) | Yes | Landen et al. 2017a |
|  | NCT00945672 | 2009-011172-30 |  | 2 | 2009 | Complete | 36 | Europe (1) |  | Landen et al. 2017b |
| Rosiglitazone | NCT00428090 |  |  | 3 | 2007 | Complete | 862 | Asia (5), Europe (9), North America (3), Oceania (1), South America (2) |  | Gold et al. 2010 |
| Semagacestat | NCT00244322 |  |  | 2 | 2005 | Complete | 51 | North America (1) |  | Fleisher et al. 2008 |
|  | NCT00594568 |  |  | 3 | 2008 | Complete | 1537 | Africa (1), Asia (3), Europe (10), North America (2), Oceania (1), South America (2) |  | Henley, Sundell, Sethuraman, Dowsett & May, 2014 |
|  | NCT00762411 |  |  | 3 | 2008 | Complete | 1108 | Asia (4), Europe (10), North America (3), South America (1) |  | - |
| Semorinemab | NCT03289143 | 2017-001800-31 |  | 2 | 2017 | Active | 457 | Europe (10), North America (2), Oceania (1) |  | - |
|  | NCT03828747 |  |  | 2 | 2019 | Recruiting | 260 | Europe (3), North America (1) |  | - |
| Sodium selenate |  |  | ACTRN12611001200976 | 2 | 2009 | Complete | 40 | Oceania (1) |  | Malpas et al. 2016 |
| Solanezumab | NCT00329082 |  |  | 2 | 2006 | Complete | 52 | North America (1) |  | Farlow et al. 2012 |
|  | NCT00905372 |  |  | 3 | 2009 | Complete | 1012 | Asia (1), North America (2), South America (2) | Yes | Doody et al. 2014 |
|  | NCT00904683 |  |  | 3 | 2009 | Complete | 1040 | Asia (3), Europe (8), North America (1), Oceania (1) | Yes | Doody et al. 2014 |
|  | NCT01148498 |  |  | 2 | 2010 | Complete | 55 | North America (1) |  | - |
|  | NCT01900665 |  |  | 3 | 2013 | Terminated | 2129 | Asia (1), Europe (7), North America (2), Oceania (1) | Yes | Honig et al. 2018 |
|  | NCT02760602 | 2016-000108-27 |  | 3 | 2016 | Terminated | 26 | Asia (2), Europe (8), North America (3) | Yes | - |
| Solanezumab / gantenerumab | NCT01760005 | 2013-000307-17 |  | 3 | 2013 | Recruiting | 490 | Asia (2), Europe (7), North America (4), Oceania (1), South America (3) |  | - |
| Tarenflurbil (r-flurbiprofen) | NCT00105547 |  |  | 3 | 2005 | Complete | 1649 | North America (1) |  | Green et al. 2009 |
|  | NCT00322036 |  |  | 3 | 2006 | Terminated | 800 | Europe (10), North America (2) |  | - |
|  |  |  |  | 2 | 2003 | Complete | 189 | Europe (1), North America (1) |  | Wilcock et al. 2008 |
| Thalidomide | NCT01094340 |  |  | 2 | 2010 | Unknown | 25 | North America (1) |  | Decourt et al. 2017 |
| Thiethylperazine | NCT03417986 |  |  | 2 | 2018 | Active | 100 | Europe (1) |  | - |
| Tideglusib | NCT01350362 |  |  | 2 | 2011 | Complete | 306 | Europe (6) |  | Lovestone et al. 2015 |
| Tilavonemab | NCT02880956 | 2016-001634-10 |  | 2 | 2016 | Active | 454 | Asia (1), Europe (7), North America (2), Oceania (2) |  | - |
| Tramiprosate (homotaurine) |  |  |  | 2 | 2002 | Complete | 58 | North America (1) |  | Aisen et al. 2006 |
|  | NCT00088673 |  |  | 3 | 2004 | Complete | 1052 | North America (2) |  | Saumier, Duong, Haine, Garceau, & Sampalis, 2009; Gauthier et al. 2009 |
| UB-311 | NCT02551809 |  |  | 2 | 2015 | Complete | 43 | Asia (1) | Yes | - |
| Vanutide cridificar (ACC-001) | NCT00479557 | 2006-002061-39 |  | 2 | 2007 | Complete | 86 | Europe (3) | Yes | Pasquier et al. 2016 |
|  | NCT00498602 |  |  | 2 | 2007 | Complete | 160 | North America (1) |  | Pasquier et al. 2016 |
|  | NCT00752232 |  |  | 2 | 2008 | Complete | 40 | Asia (1) |  | Arai, Suzuki, & Yoshiyama (2015) |
|  | NCT00959192 |  |  | 2 | 2009 | Complete | 32 | Asia (1) |  | Arai, Suzuki, & Yoshiyama (2015) |
|  | NCT01227564 |  |  | 2 | 2010 | Complete | 63 | North America (1) |  | Van Dyck et al. 2016 |
|  | NCT01284387 |  |  | 2 | 2011 | Complete | 126 | North America (1) |  | Ketter et al. 2016 |
| Varoglutamstat (PQ912) | NCT02389413 | 2014-001967-11 |  | 2 | 2015 | Complete | 120 | Europe (7) |  | Scheltens et al. 2018 |
| Verubecestat | NCT01739348 | 2011-003151-20 |  | 3 | 2011 | Terminated | 1958 | Asia (3), Europe (12), North America (2), Oceania (2), South America (2) |  | Egan et al. 2018 |
|  | NCT01953601 | 2012-005542-38 |  | 3 | 2012 | Terminated | 1454 | Africa (1), Asia (2), Europe (13), North America (2), Oceania (2), South America (2) |  | Egan et al. 2019 |
| Zagotenemab | NCT03518073 |  |  | 2 | 2018 | Active | 285 | Asia (1), North America (2) |  | - |
| * Central American countries were included in North America. Russia was categorized as European. Puerto Rico is a US territory, however we counted it as a separate North American country for the purpose of this table | | | | | | | | | | |

| **Supplementary Table 2. Median percent white for trials with and without specific eligibility criteria** | | |
| --- | --- | --- |
| **Criterion** | **Used as criterion, median %white (n trials)** | **Not used as criterion,  median %white (n trials)** |
| **Medical conditions** |  |  |
| Other neurological disease | 95.80% (35) | 92.50% (11) |
| Psychiatric disorder | 95.45% (38) | 88.10% (8) |
| Cardiovascular disease | 94.50% (31) | 96.10% (15) |
| Cerebrovascular disease | 96.15% (30) | 87.15% (16) |
| - Hachinski ischemia scale score >4 | 94.30% (30) | 96.25% (16) |
| - Cerebrovascular evidence on MRI | 95.45% (24) | 94.20% (22) |
| Childbearing/conception | 91.40% (27) | 96.30% (19) |
| Unspecified systemic illness | 95.95% (32) | 91.95% (14) |
| Alcohol or drug abuse | 91.95% (22) | 95.45% (24) |
| Vitals or lab abnormalities | 94.80% (23) | 94.50% (23) |
| Infections/infectious diseases | 90.60% (23) | 96.30% (23) |
| - HIV status | 83.70% (11) | 95.80% (35) |
| Liver disease | 92.50% (21) | 95.10% (25) |
| Autoimmune disease | 95.45% (26) | 94.20% (20) |
| Renal disease | 92.50% (17) | 95.10% (29) |
| Seizure disorder | 95.80% (23) | 94.10% (23) |
| Cancer | 95.10% (21) | 94.30% (25) |
| Respiratory illness | 95.10% (11) | 94.50% (35) |
| Endocrine dysfunction | 92.80% (12) | 95.45% (34) |
| Brain/head trauma | 90.60% (15) | 96.20% (31) |
| Diabetes | 94.80% (9) | 94.50% (37) |
| Weight or BMI cut-off | 95.95% (6) | 94.20% (40) |
| Gastrointestinal disease | 94.50% (9) | 94.80% (37) |
| CNS inflammation | 96.25% (8) | 94.40% (38) |
|  |  |  |
| **Undergoing study procedures** |  |  |
| Caregiver attendance | 94.10% (39) | 96.30% (7) |
| Written informed consent | 94.50% (31) | 96.10% (15) |
| Contraindication to MRI/PET | 94.95% (22) | 94.40% (24) |
| Adequate sensory abilities | 94.95% (20) | 94.20% (26) |
| Language ability | 96.15% (20) | 91.95% (26) |
| Residence in the community | 96.20% (19) | 90.60% (27) |
| Caregiver consent | 95.10% (21) | 90.60% (25) |
| Education requirement | 96.25% (8) | 94.20% (38) |
| Reading or writing ability | 95.35% (12) | 94.55% (34) |
| Determined likely to complete | 95.10% (9) | 94.50% (37) |
|  |  |  |
| **Cognitive and neuropsychiatric measures** |  |  |
| CDR | 95.45% (8) | 91.25% (38) |
| Geriatric Depression Scale | 87.15% (12) | 94.95% (34) |
| Hamilton Depression Rating Scale | 95.10% (6) | 94.65% (40) |
| * The following variables were excluded because ≤5 studies reporting ethnicity data used this criterion: ‘systemic inflammation’, ‘excessive smoking’, ‘Recent hospitalization’, ‘other depression instrument’, ‘CSSRS’, ‘other suicide/self-harm risk scale’, ‘MoCA’, ‘Eastern Cooperative Oncology Group Status’, ‘FAQ’, ‘ADAS-Cog’, ‘RBANS’, ‘Memory-specific test’. Similarly, the MMSE was removed as ≤5 studies reported not using this criterion. | | |

**Supplementary Text 1**

Review search strategy

**embase.com**

('Alzheimer disease'/de OR 'dementia'/de OR 'mild cognitive impairment'/de OR (Alzheimer* OR dementia* OR (mild* NEAR/3 cogniti* NEAR/3 (impair*))):ab,ti) AND ('drug therapy'/de OR 'Alzheimer disease'/de/dm_dt OR psychopharmacotherapy/de OR 'psychotropic agent'/exp OR 'immunotherapy'/exp OR 'amyloid beta protein'/de OR 'enzyme inhibitor'/exp OR (drug* OR agent* OR psychopharmacotherap* OR pharmac OR inhibitor* OR (monoclonal* NEAR/3 antibod*) OR immunotherap* OR immun*-therap* OR amyloid-β OR β-amyloid OR beta-amyloid OR aβ OR a-β OR amyloidβ OR amyloid-beta):ab,ti) AND ('phase 2 clinical trial'/exp OR 'phase 3 clinical trial'/exp OR ((study OR trial*) NEAR/10 (phase-2 OR phase-2a OR phase-2b OR phase-2-a OR phase-2-b OR phase-3 OR phase-ii OR phase-iia OR phase-iib OR phase-ii-a OR phase-ii-b OR phase-iii)):ab,ti) NOT ([Conference Abstract]/lim) NOT ([animals]/lim NOT [humans]/lim) NOT ([Conference Abstract]/lim) AND [english]/lim NOT ('systematic review'/de OR 'meta analysis'/de OR ((systematic NEAR/3 review*) OR meta-analys* OR metaanalys*):ti)

**Medline Ovid**

(Alzheimer Disease/ OR Dementia/ OR (Alzheimer* OR dementia* OR (mild* ADJ3 cogniti* ADJ3 (impair*))).ab,ti.) AND (drug therapy/ OR Alzheimer Disease/dt OR exp Psychotropic Drugs/ OR exp Immunotherapy/ OR exp Amyloid beta-Peptides/ OR exp Enzyme Inhibitors/ OR (drug* OR agent* OR psychopharmacotherap* OR pharmac OR inhibitor* OR (monoclonal* ADJ3 antibod*) OR immunotherap* OR immun*-therap* OR beta-amyloid OR amyloid-beta).ab,ti.) AND (Clinical Trial, Phase II/ OR Clinical Trial, Phase III/ OR ((study OR trial*) ADJ10 (phase-2 OR phase-2a OR phase-2b OR phase-2-a OR phase-2-b OR phase-3 OR phase-ii OR phase-iia OR phase-iib OR phase-ii-a OR phase-ii-b OR phase-iii)).ab,ti.) NOT (news OR congres* OR abstract* OR book* OR chapter* OR dissertation abstract*).pt. NOT (exp animals/ NOT humans/) NOT (news OR congres* OR abstract* OR book* OR chapter* OR dissertation abstract*).pt. AND english.la. *NOT (Systematic Review/ OR Meta-Analysis/ OR ((systematic ADJ3 review*) OR meta-analys* OR metaanalys*).ti.)*

**Cochrane CENTRAL**

((Alzheimer* OR dementia* OR (mild* NEAR/3 cogniti* NEAR/3 (impair*))):ab,ti) AND ((drug* OR agent* OR psychopharmacotherap* OR pharmac OR inhibitor* OR (monoclonal* NEAR/3 antibod*) OR immunotherap* OR immun* next therap* OR amyloid next β OR β next amyloid OR beta next amyloid OR aβ OR a next β OR amyloidβ OR amyloid next beta OR *mab):ab,ti) AND ((phase next 2 OR phase next 2a OR phase next 2b OR phase next 2 next a OR phase next 2 next b OR phase next 3 OR phase next ii OR phase next iia OR phase next iib OR phase next ii next a OR phase next ii next b OR phase next iii):ab,ti)

**Clinicaltrials.gov**

Condition or disease: Alzheimer OR dementia

Other terms: antibodies OR inflammatory OR "rage antagonists" OR "calcium channel" OR amyloid OR tau OR psychotropics OR statins OR "hmg coa"

Additional Criteria: Phase: Phase 2 OR Phase 3

**Supplementary Text 2**

*Analyses of studies with and without a full protocol available*

We compared studies with and without a full protocol using Fisher’s Exact test (without correcting for multiple testing). Studies for which a full protocol was available more often contained criteria related to childbearing/conception (p<.001), a cardiovascular disease history (p<.05), Hachinski scores (p<.05), brain trauma (p=.01), respiratory illness (p<.05), infection (p<.001), HIV-status (p<.05), autoimmune disease (p=.001), cancer (p<.001), vital and lab abnormalities (p<.05), a contraindication to undergoing an MRI scan (p<.01), sensory abilities (p<.01), written informed consent (p<.05), language (p<.05), caregiver consent (p<.05), and whether patients are likely to complete the study (p<.001).
